# Do índio Passos ao doutor Chernoviz: experiências de cura da lepra no Pará do século XIX

**DOI:** 10.1590/S0104-59702023000100027

**Published:** 2023-08-04

**Authors:** Márcio Couto Henrique

**Affiliations:** i Faculdade de História Universidade Federal do Pará Belém PA Brasil marciocouto@ufpa.br Professor, Faculdade de História/Universidade Federal do Pará. Belém – PA – Brasil. orcid.org/0000-0002-0170-5315. marciocouto@ufpa.br

**Keywords:** Lepra, Medicina, Protagonismo indígena, Amazônia

## Abstract

O artigo analisa uma experiência de cura da lepra com assacu (*Hura crepitans* L.), realizada em Santarém, Pará, em 1847, por um indígena chamado Antonio Vieira dos Passos. A experiência passou a ser realizada nas demais províncias do Brasil e também no exterior. Por essa razão, o artigo estabelece relações com práticas médicas realizadas em outras partes do país, tendo como foco o diálogo entre a medicina oficial e a medicina indígena. A análise de matérias de jornais e documentos oficiais revelou que os saberes indígenas sobre o uso de plantas medicinais eram amplamente reconhecidos e utilizados pelos médicos com a intenção de incorporá-los em seu repertório terapêutico.

## A beleza do morto

Quando viu sua pele coberta de tubérculos, dedos das mãos atrofiados e rosto deformado, Marianno José Machado, de 50 anos de idade, passou a residir no Hospital dos Lázaros do Rio de Janeiro. O estigma (Goffman, 1975)^1^ que acompanhava a doença, então chamada de lepra, tornava impossível sua convivência em sociedade.^2^ Desgostoso da vida, Marianno decidiu se submeter a uma arriscada experiência que, diziam alguns, poderia livrá-lo daquela terrível doença: ser picado por uma cobra cascavel. Após assinar declaração assumindo para si a responsabilidade, o leproso introduziu a mão direita na gaiola em que se encontrava o animal, que não tardou a picar seu dedo.

Matéria publicada dez anos depois informava que Marianno decidiu se submeter a essa experiência “apesar dos prudentes conselhos de muitos médicos que duvidavam do bom processo desse perigoso meio” (Extrato da viagem..., 25 fev. 1848, p.3). O certo é que ninguém tentou proibir a experiência, e Marianno, “até a meia noite, sofreu cruelmente”. Os poucos remédios que lhe foram ministrados pelos médicos presentes em nada diminuíram seu sofrimento, e a matéria revela a postura de passiva contemplação da plateia, desde a picada da cobra, às 11h50 da manhã do dia 4 de setembro de 1838, até 11h30 do dia seguinte, quando Marianno “deu alma a Deus”. Movidos pela curiosidade científica e indiferentes à situação do doente, os assistentes registraram, minuto a minuto, as reações do leproso, o que indica que ele era instado a relatar tudo o que sentia: o sangue escorrendo na região picada, o inchaço da mão, frio, alteração da vista, formigamento no rosto, ansiedade, fechamento da garganta, dores no peito, pulsação irregular, até o momento em que faleceu (Extrato da viagem..., 25 fev. 1848, p.3). O médico francês Xavier Sigaud (2009, p.271) registrou, em 1844, a frustração final que os médicos sentiram um dia após a morte do leproso: “O odor fétido que se desprende às 10 horas da manhã do dia seguinte é tal que impede a realização da autópsia, para grande decepção das pessoas da arte”.

Experiências menos trágicas de cura da lepra eram realizadas por todo o Brasil ao longo do século XIX, e os jornais da época frequentemente anunciavam supostas curas da doença, tanto no Brasil como no exterior. Em 1844, anunciava-se a cura da “horrível moléstia da elefancia”, que teria sido descoberta por uma portuguesa na cidade do Porto, Portugal (Cura da lepra, 9 mar. 1844, p.3). Em 1848, ganhou notícia a descoberta de um “meio de curar radicalmente a morfeia, com ervas e outros específicos” na vila de Itapetininga, São Paulo (Saúde pública, 18 ago. 1848, p.5). O descobridor era um francês chamado Charles Pierre Etéchéion, que, desde 1847, fazia experiências de cura da lepra utilizando-se do guano, material constituído das fezes de aves e morcegos (Cabral, 2007). Em 1859, em um pequeno povoado chamado Paracari, próximo a Santarém, no Pará, o curandeiro Antonio Francisco da Costa se dizia capaz de curar a lepra com um remédio ao qual ele deu o mesmo nome do lugar, Paracari (Instruções..., 30 jun. 1859, p.1).

Matéria publicada no jornal *Treze de Maio*, em 1840, aponta para o imaginário da época em torno da doença, o crescimento de casos de lepra na província do Pará e a esperança que esse tipo de notícia gerava:

Não há pessoa que não conheça o terrível e progressivo flagelo, que de dia em dia se vai tornando mais ameaçador para a nossa província: a elefantíase! Nós todos sentimos a urgente necessidade que há de tomarmos as mais sérias providências e cautelas, para que um semelhante mal não se derrame pela população, se generalize e afinal só tenhamos de recriminar nossa apatia (Projeto, 2 out. 1840, p.146).

A lepra era, então, vista como um “terrível flagelo”, ameaça para a vida em sociedade, mal a ser extirpado da província ou, pelo menos, posto sob controle. Para isso, o articulista defendia que o governo provincial construísse um lazareto em lugar afastado e promovesse o envio anual de leprosos para a província de Goiás, “a fim de usarem das Caldas” (Projeto, 2 out. 1840, p.146). As águas de Caldas Novas, em Goiás, assim como as de Poços de Caldas, no sul de Minas, eram consideradas auxiliares eficientes na cura de doenças como lepra, sífilis e moléstias venéreas em geral. Muitas pessoas desenganadas com os recursos ordinários possíveis nas vilas e cidades nutriam esperança no poder daquelas águas tidas como virtuosas (Marras, 2004). A doença também era conhecida como morfeia, gafa, elefantíase dos gregos, elefancia, mal de lázaro, mal da pele e mal do sangue. Lepra, então, era sinônimo de tudo que se considerava repugnante, que poderia corromper a moralidade, destruir a vida social, abalar a ordem natural das coisas.

Em junho de 1847, os moradores de Belém do Pará leram no jornal *Treze de Maio* a notícia de que, em Santarém, havia sido descoberto um remédio para a cura da lepra.^3^ Ao procurar informações sobre o fato, o vice-presidente da província, João Maria de Moraes, soube que certo José Joaquim de Souza Gomes, de longa data conhecido como leproso, teria aparecido curado depois de tratado com aplicações de assacu (*Hura crepitans* L.) por um indígena chamado Antonio Vieira dos Passos (Pará, 1847, p.19). O médico polonês Chernoviz, em 1842, fez a seguinte descrição do assacu:

Árvore colossal, de folhas subcordiformes, ovais, denteadas; fruto, cápsula lenhosa, multicoca, com uma semente em cada loculamento. Extrai-se por incisão dessa árvore um suco gomoso branco-pardacento ou branco-avermelhado, que se condensa e solidifica com dificuldade e vagar; quando condensado, é escuro-pardacento, com o aspecto mais de goma que de resina, e mui solúvel em água (Chernoviz, 1878, p.254-255).

Até então, sabia-se do uso do assacu pelos índios tal qual havia sido descrito por Martius (1854, p.167): “o suco lácteo dessa árvore equatorial é empregado pelos índios como anti-helmíntico e para embriagar os peixes”, apanhando-os, depois, com a mão.

Naquela ocasião, Passos se encontrava preso, em Santarém, acusado de homicídio. Imediatamente, a Câmara Municipal da cidade determinou a realização de experiências com aplicação do assacu em cinco leprosos, sob a observação do cirurgião prático da vila, Raymundo José Rebello. Este fez chamar à sua presença o índio Antonio Vieira dos Passos e o inquiriu, em Santarém, definindo-o como “de espírito desembaraçado e enérgico, posto que rústico, pouco ou nada lê, pode assinar, contudo, o seu nome” (Vila de Santarém..., 21 set. 1847, p.2). Passos não se intimidou diante do cirurgião:

Afirma com regularidade que não teme curar qualquer morfético sob as vistas de médicos. Não deixa, porém, de exprimir-se assim: que quem tem de morrer da moléstia sempre morre, quiçá querendo dizer que assim como outras moléstias, que têm remédios conhecidos, nem sempre com eles se curam, também não é infalível o seu remédio da morfeia. Diz ele que nem todos os remédios que aplica tem declarado. Todavia, parece que todos ou o essencial, possui o cirurgião Rebello (Vila de Santarém..., 21 set. 1847, p.2).

O relato é importante porque, ainda que de modo indireto, revela a fala do índio Passos, sua segurança na defesa do método utilizado para a cura da lepra e a leitura comparada que ele fazia entre sua prática de cura e a da medicina oficial, ambas falíveis ou incapazes de curar em todos os casos. Ao mesmo tempo, o relato sugere que Passos se esforçava em guardar para si certo controle do uso dos medicamentos que utilizava, não declarando todos de que fazia uso. Quatro meses depois de iniciada a experiência em Santarém, o cirurgião informava que os doentes apresentavam sensíveis melhoras e que havia esperança de curá-los completamente com mais três meses de tratamento.

Vários jornais publicaram o receituário utilizado pelo índio Passos:

O Sr. Rebello diz que a forma por que ele emprega o assacu no curativo dos morféticos em Santarém é a de que serviu Souza Gomes no tratamento da sua moléstia e que somente a aperfeiçoou e a adaptou às regras da Ciência Médica. A forma é assentada no uso diário de pílulas feitas do suco inspissado (que tem recebido o nome de extrato); no uso da mistura composta de meia libra de cozimento forte da casca e de dez a vinte gotas do suco (a qual tem o nome de vomitório); o enfermo é obrigado a beber de uma só vez toda a porção da mistura com o fim de fazer-se vomitar e assim é por que os doentes vomitam para mais de seis vezes. Desse meio é repetido o uso de oito em oito dias. No uso de banhos gerais preparados pelo cozimento saturado da casca e repetidos de dias a dias; e no uso de um cozimento emoliente para bebida ordinária (Interior, 22 fev. 1848, p.3).

Note-se que o cirurgião Rebello procurava distinguir seu procedimento daquele que era utilizado pelo índio Passos, afirmando que “aperfeiçoou” e “adaptou” o método “às regras da Ciência Médica”. Essa roupagem científica visava conferir mais credibilidade e legitimidade ao tratamento experimental.

Matéria publicada no jornal *Diário do Rio de Janeiro* informava o recebimento de carta oriunda de Santarém, a qual dizia que “parece que com um veneno denominado assacu e que produz o Pará, aplicado em vomitório e em purgante de um modo ensinado por um índio dos Parintins, se cura perfeitamente a morfeia” (Cura da morfeia, 5 ago. 1847, p.3). Essa foi a única publicação sobre o caso em que encontrei referência ao possível pertencimento étnico de Antonio Vieira dos Passos. No século XVII, missionários jesuítas fundaram a missão de Tupinambaranas, origem remota da atual cidade de Parintins. Em 1803, índios maué e munduruku foram reunidos no local. Parintins, nome oficializado em 1880, seria uma referência aos parintintins que habitavam a região. Em 1832, Baena (2004, p.340; publicado originalmente em 1839) indicava uma população de 970 índios no lugar, que então se chamava Vila Nova da Rainha. Assim, a indicação de que o remédio para a cura da lepra teria sido descoberto por “um índio dos Parintins” é uma afirmação genérica, de modo que Antonio Vieira dos Passos poderia pertencer a qualquer um dos povos indígenas que habitava a região. De todo modo, é importante chamar atenção para essa informação, pois a identidade étnica ou mesmo os nomes dos índios que forneceram informações ou revelaram plantas de uso medicinal para a medicina dita científica são dados tradicionalmente apagados, a exemplo da erva intitulada aiapana (*Ayapana triplinervis* (M. Vahl) R.M.King e H. Rob.), revelada por uma mulher indígena de nome e etnia desconhecidos, mas que se tornou conhecida como a “erva-do-ouvidor”, em referência à autoridade de Belém cujo único mérito foi enviar a planta para Lisboa, com a indicação de suas virtudes terapêuticas (Sanjad, Pataca, Santos, 2021). A ayapana é eficiente no tratamento de desordens gastrointestinais, afecções da boca, febre, verminoses, além de ser utilizada como sudorífero e antídoto para mordeduras de cobras.

Ao longo do século XIX, médicos e estudiosos se mostravam especialmente atentos aos remédios utilizados pelos índios. Das viagens de Karl F.P. von Martius pelo Brasil, entre 1817 e 1820, resultou, entre outras, a obra *Natureza, doenças, medicina e remédios dos índios brasileiros* (Martius, 1979), publicada originalmente em 1844. No capítulo intitulado “Medicamentos do reino vegetal”, Martius (1979, p.152) afirma que “essas plantas medicinais têm, além disso, no estado fresco em que são empregadas pelo médico selvagem, a mais eficaz virtude medicamentosa e em muitos casos substituem, com feliz êxito, as composições químicas da medicina europeia”. Apesar de considerar que o conhecimento do “médico selvagem” é “uma face do mais grosseiro empirismo”, o autor dizia que essa matéria “merece toda atenção da medicina racional”. Assim, por um lado, Martius hierarquizava os saberes, negando aos índios o conhecimento racional dos efeitos curativos das plantas medicinais das quais faziam uso.^4^ Por outro, considerava que esse mesmo conhecimento merecia a atenção da ciência dita racional. Certamente, a posição do autor estava relacionada com a imagem que ele tinha dos índios brasileiros, vistos por ele como povos degenerados e sujeitos a extinção em breve (Martius, 1844). Apesar de sua desconfiança, Martius (1979, p.152) relatou ter presenciado diversas curas realizadas por “médicos indígenas”, algumas delas de efeito tão rápido e eficaz que “atingiu as raias do maravilhoso”, como se dissesse que as “plantas míticas” utilizadas pelos índios, mais do que curar, faziam milagres. De fato, o autor dizia que, como era impossível afirmar como elas foram descobertas pelos índios, e, devido ao fato de que, em torno delas, geralmente existe um mito explicativo de tal origem, “a respeito dessas plantas devemos admitir que provieram do Paraíso” (1979, p.155).

Matéria publicada no Rio de Janeiro a respeito do assacu revela esse misto de descrédito e curiosidade pela medicina indígena:

Os médicos do Pará estão falando muito em um descobrimento assaz importante para a ciência, devido à prática dos índios. Dizem que a morfeia se cura excelentemente com o assacu, euforbiacea denominada pelo Dr. Martius Ura Brasiliensis. Igual virtude e antissifilítica se atribui a vários outros vegetais do Pará, manacá, mururé, cururu-timbó etc.

... Alguém há, porém, que ponha em dúvida essa apregoada virtude e lembrados da sorte que já, em caso idêntico, teve o guano, esperamos o resultado de novas experiências, sem que, todavia, nos esqueçamos que as virtudes de várias ipecacuanhas e outras plantas nos foram ensinadas pelos índios (Os médicos do Pará..., 1848, p.125-126).

Note-se que o articulista atribuía o descobrimento à “prática dos índios”, assim como Martius atribuía o saber indígena ao “mais grosseiro empirismo”. E, aos que colocavam em dúvida a “apregoada virtude” dos índios no descobrimento de plantas medicinais, o articulista lembrava o exemplo da ipecacuanha e de outras plantas de uso curativo que “nos foram ensinadas pelos índios”.^5^ Eis o paradoxo diante de povos considerados degenerados, em vias de extinção, mas dos quais a ciência ocidental poderia obter importantes contribuições, antes que essa “sabedoria natural, ora decadente”, desaparecesse de vez. Era necessário, portanto, apropriar-se do “saber natural” dos índios, tido como resquício de um tempo de glória situado em um passado mítico, do qual os índios do século XIX seriam uma pálida representação. Urgia, então, apropriar-se da “beleza do morto” (Certeau, 1995), traduzindo o saber indígena em uma linguagem científica que neutralizasse seus supostos perigos. Era preciso comprovar cientificamente os efeitos do assacu, realizando testes, experiências, provas e contraprovas, sem as quais o conhecimento era classificado como meramente empírico, não científico, incapaz de comprovar a eficácia farmacológica de uma planta.

Segundo publicação do *Arquivo Médico Brasileiro*, “não há dúvida que o Pará, por amor do luxo e magnificência de sua vegetação, fornece a todos os males físicos uma série de plantas salutares ... Não é, pois, de admirar que se leiam nos últimos números dos jornais dessa província curas da lepra pelo emprego do veneno assacu e da tísica pulmonar pelo uso da casca de mata-matá” (Remédios..., jul. 1847, p.244). Ao destacar a “magnificência de sua vegetação”, o articulista reforça a ideia de uma “sabedoria natural” dos índios, vistos, eles próprios, como parte da natureza, segundo a concepção do “índio ecológico” (Duarte, 2005). De todo modo, notícias como essa, quando vindas do Pará, mereciam atenção dos médicos. Dizia Sigaud (2009, p.131) que o Pará “é a mais rica de todas as províncias do Brasil em plantas alimentícias e especiarias”. Em 1859, o doutor Francisco da Silva Castro, um dos mais conceituados médicos de Belém, reuniu outros médicos em sua casa “para ouvirem ler uma obra de sua composição, intitulada Enumeração dos vegetais indígenas do Brasil empregados em Medicina e mais usados, sua ação, doses, fórmulas etc.” e que faria parte do compêndio de matéria médica do senhor doutor Beirão, um médico português (Consta-nos que o Sr..., 4 set. 1859, p.2).

Compreende-se, assim, a curiosidade dos médicos em torno da suposta descoberta da cura da lepra pelo índio Passos. Em ofício enviado por João Baptista Gonçalves Campos, juiz de direito da comarca de Santarém, ao presidente da província do Pará, Herculano Ferreira Pena, Campos narrou como foi seu encontro com “o pardo Antonio Vieira dos Passos”, que confirmou ter curado José Joaquim de Souza Gomes:

Declarou mais que já conseguiu curar perfeitamente a um tapuio de nome Theodozio, do Corpo de Trabalhadores de Faro, o qual se achara miseravelmente ferido do mal de morfeia, mas que hoje bom trabalha por seu ofício de carpina naquela vila. Que aprendeu este método de cura de um curibocolo velho da freguesia de Juruti chamado Manoel Joaquim, também do Corpo de Trabalhadores, mas hoje falecido (Vila de Santarém..., 21 set. 1847, p.2).

A informação de que Antonio Vieira dos Passos teria aprendido seu método de cura com um “curibocolo velho da freguesia de Juruti” revela que o uso do assacu para o tratamento da lepra era bem mais antigo entre os índios. “Curibocolo” é uma corruptela de “curiboca” ou “caraiboca”, termo que, segundo o *Dicionário de palavras brasileiras de origem indígena*, significa “caboclo”, “filho de índio com negro”, “mestiço de branco e índio” (Chiaradia, 2008, p.166). Anos depois, Mello Moraes (1881, p.58-59) dizia que Antonio Vieira dos Passos, “para esse curativo, usava do leite do assacu, e que esse remédio lhe tinha ensinado um caboclo de Juruti, do Pará, chamado Manoel José Joaquim”. Na Amazônia do século XIX, depois de batizados e de aprenderem rudimentos da língua portuguesa, os índios tinham sua identidade étnica negada, sendo chamados de caboclos ou tapuios (Harris, 1998; Henrique, 2018). De acordo com Baena (2004, p.235), viviam em Juruti, em 1832, 385 indígenas munduruku e maué. Assim, fica evidente que o assacu era utilizado em experiências de cura da lepra por índios dessa região, no oeste do Pará, em lugares como Parintins e Juruti. Por outro lado, fica claro que, distantes do saber médico das cidades, pessoas como o “tapuio Theodózio”, de Faro, ou a preta Eusébia, de Vila Franca, “que está, também, curada da mesma enfermidade” (Tendo chegado..., 16 out. 1847, p.2), recorriam aos pajés para a cura de suas doenças.

Em 1840, João Antonio de Miranda, presidente da província do Pará, convocou alguns médicos para dar parecer acerca de um grupo de pessoas apresentadas como curadas da lepra pelo cirurgião da Armada, Marcelo Domingues Barbosa. Os médicos Camillo José do Valle Guimarães, Francisco da Silva Castro, José Custódio da Fonseca Paes e Alexandre da Costa Araújo trataram de desqualificar o curandeiro, classificando-o como charlatão e dizendo que alguns dos quais se diziam curados da lepra nunca foram leprosos. Diziam, ainda, que ele fazia uso particular de remédios conhecidos da medicina, juntamente com “extravagantes composições” que incluíam as plantas chamadas de mururé (*Brosimum acutifolium* Huber),^6^ marapuama (*Ptychopetalum olacoides* Bentham) e o assacu (Elefantíasis, 4 jul. 1840, p.72). Observa-se, então, que o assacu era utilizado em experiências de cura da lepra por curandeiros antes do índio Passos.

Segundo Gilberto Freyre (1946, p.598), “médicos e curandeiros nunca estiveram muito distanciados uns dos outros, antes da segunda metade do século XIX”. Plantas mal estudadas ou desconhecidas pela medicina oficial eram amplamente utilizadas em experiências de cura das mais diversas doenças. A respeito das práticas terapêuticas utilizadas por Antônio Corrêa de Lacerda e Francisco da Silva Castro, Sanjad e Costa (2019, p.57) afirmam: “Pode-se observar uma abertura para experimentos baseados em conhecimentos populares e indígenas, sobretudo, no uso de plantas medicinais. Esse traço parece ter sido comum entre os médicos que atuaram na região amazônica antes do surgimento da bacteriologia e da medicina dita científica”. Assim, os médicos reproduziam a conduta que costumavam criticar nos índios, fazendo uso de um arsenal farmacológico totalmente experimental e empírico, ancorado em usos e costumes locais. Naquele contexto, a fronteira que separava a medicina de outras práticas de cura ainda não era bem definida, ensejando entre os médicos das províncias brasileiras uma vasta experimentação de práticas de cura de origem popular, conforme assinalam os inventários etnobotânicos que registravam qualquer conhecimento que pudesse ter aplicação na medicina e na indústria (Sampaio, 2001; Guimarães, 2016).

## Da aldeia para o mundo

Em outubro de 1847, José de Souza Gomes, o morador de Santarém que se dizia curado da lepra graças ao tratamento do índio Antonio Vieira Passos, chegou a Belém, onde foi examinado no Palácio do Governo por uma junta médica composta pelos doutores Camillo José do Valle Guimarães, José da Gama Malcher e Joaquim Frutuoso Guimarães. No relatório apresentado à presidência da província, constam algumas informações que nos permitem conhecer um pouco de sua biografia. Diziam os médicos que José de Souza Gomes era filho de Antonio de Souza Gomes, nascido em Belém do Pará e que estava com 33 anos. Era solteiro, “mameluco, de temperamento linfático, de constituição relativa a este temperamento; tem sido desregrado em seu modo de viver; teve bexigas e a moléstia sifilítica” (Interior, 22 fev. 1848, p.2). Três anos antes, havia sido internado no Hospital de Caridade, em Belém, a fim de se tratar de uma doença de pele que ele desconhecia que fosse lepra, sendo orientado pelo doutor José da Gama Malcher a se recolher no leprosário do Tucunduba. Insatisfeito com essa determinação, Gomes fugiu do Hospital da Caridade e se retirou para o interior da província “em procura da morte”, diziam os médicos. Ocorreu que

ali um sujeito lhe propusera a cura da sua enfermidade por meio do assacu; desgostoso o paciente do hediondo estado em que se via aceitou a oferta da cura; duvidoso, porém, do que se lhe prometia, esperava que este meio, como veneno, encurtasse os dias da sua vida, esperança que ele tinha como lenitivo aos seus males; contudo, não sucedeu assim, o acaso lhe deparou este remédio que modificou a sua enfermidade a ponto que o tem conduzido a poder voltar para o grêmio da sociedade, de onde fora sequestrado (Interior, 22 fev. 1848, p.2).

Em seguida, os médicos trataram de apresentar o quadro em que Gomes se encontrava quando foi examinado pelo doutor Malcher no Hospital da Caridade, antes de sua fuga para o interior da província. Os médicos descreveram sintomas típicos de uma pessoa acometida pela lepra, tais como hálito fétido, a voz rouca, o rosto inchado, fosco e rugoso e que “causava repugnância pela deformidade de suas feições”, as pernas inchadas, dedos das mãos deformados (Interior, 22 fev. 1848, p.2). A descrição dos sintomas da lepra contribuía para reforçar o estigma em torno da doença e, especialmente, do doente. Gomes foi posto nu e observado minuciosamente pela junta médica em um intervalo de pouco mais de dois meses. Os médicos chegaram à conclusão de que “a mudança que apresentam a face, o tronco e os membros torácicos que se achavam atacados da lepra tuberculosa é agradável aos olhos do médico, porque ela dá toda a esperança de que, se o enfermo Souza Gomes insistir no uso dos meios de que tem tirado proveito, há de chegar ao completo restabelecimento da sua saúde” (Interior, 22 fev. 1848, p.3).

Em outubro de 1848, o índio Passos chegou à capital paraense, sendo recebido pelo presidente da província, o chefe de polícia, um médico da Câmara e outro da Santa Casa. O presidente Jerônimo Francisco Coelho tentou, então, convencê-lo a declarar o processo de fabricação e uso do assacu, prometendo remunerá-lo, ao que o índio Passos acedeu, embora continuasse “na suposição de que lhe querem arrebatar um segredo, de que ele se julga o depositário, e de que vai fazendo aplicação empírica” (Pará, 1848, p.96). Desde os tempos coloniais, tanto em Portugal como no Brasil, era comum os “descobridores” de curas para doenças até então incuráveis guardarem para si suas fórmulas, configurando o que se convencionou chamar “remédios de segredo”. Entre os “segredistas”, encontravam-se médicos, cirurgiões, boticários, curandeiros e, também, pessoas alheias às práticas de curar (Marques, 1997). Por outro lado, os “segredistas”, “descobridores e os propagadores de remédios miríficos” (Santos Filho, 1977, p.356) eram geralmente classificados como charlatães pelos representantes da medicina oficial. De acordo com Gabriela dos Reis Sampaio (2001), charlatanismo era uma categoria abrangente, utilizada por intelectuais médicos para qualificar toda e qualquer medicina diferente da sua, desde curandeiros, espíritas, boticários, além de homeopatas e médicos estrangeiros cujos diplomas não tivessem sido convalidados pelas faculdades de medicina do país. Diz a autora que o esforço dos médicos se dava no sentido de usar as armas das quais dispunham “para não naufragar nesse mar de medicinas – e conseguir estabelecer sua prática como hegemônica” (p.53). De todo modo, Sampaio demonstra como o interior da classe médica era marcado por conflitos e contradições.^7^

Depois disso, o presidente da província autorizou o estabelecimento de uma enfermaria exclusivamente para realizar experiências com o assacu junto aos leprosos do Tucunduba (Pará, 1849, p.60). Desde sua fundação, em 1815, esse leprosário foi administrado pela Santa Casa de Misericórdia do Pará, e, por todo o século XIX, a maioria dos doentes ali recolhidos era de ex-escravizados, libertados por seus senhores após manifestar os primeiros sinais da lepra. Entre os 68 enfermos no Tucunduba em 1848, 64 eram identificados como “pessoas de cor”. Dessas, 51 eram identificadas como escravos e nove como libertos (Henrique, 2012).

A primeira dificuldade foi encontrar local adequado para instalar o “lazareto experimental” onde seis leprosos escolhidos no Tucunduba seriam submetidos a tais experiências. Dizia José Pio de Araújo Nobre, provedor da Santa Casa, que “não nos tem sido possível conseguir, porque ninguém as quer alugar para semelhante fim” (Ofícios..., 20 dez. 1848), fato que reforça o estigma a que estavam sujeitos os doentes de lepra nesse período. Por essa razão, a Santa Casa acabou utilizando prédio do governo, situado na chamada rua do Atalaia, atual travessa Joaquim Távora, em Belém. Depois das obras de adaptação, o provedor informou ao presidente da província que o local estava pronto para receber “os lázaros que vão entrar no curativo do Assacu, pelo método do Índio Passos” (Ofícios..., 13 abr. 1849).

O presidente da província determinou, então, que o doutor Camillo Guimarães fosse ao lazareto do Tucunduba e escolhesse “4 enfermos perfeitamente caracterizados como leprosos, a fim de se entrar nos ensaios e experiências do curativo” (Ofícios..., 13 abr. 1849). A preocupação em escolher “4 enfermos perfeitamente caracterizados como leprosos” estava ligada às incertezas que acompanhavam o diagnóstico da lepra naquele período.^8^ O processo de nomeação e descrição da lepra na primeira metade do século XIX era complexo e sutil, sujeito a erros e falsas interpretações, questões que “serviram para obscurecer e confundir o diagnóstico da lepra, e, ao mesmo tempo, serviram para agravar a já carregada ressonância simbólica da doença, bem como a força correspondente e o poder de suas inúmeras representações” (Robertson, 2003, p.15). Diagnósticos apressados conduziam ao recolhimento no lazareto de pessoas que não tinham lepra. Ao comentar as taxas de mortalidade no Hospício do Tucunduba entre 1844 e 1849, o presidente Jerônimo Francisco Coelho afirmou que “os lázaros pioraram pelo lado da mortalidade, agora elevada a 39 por 100, de 33 que fora no ano anterior. Os 5 que vão como curados não eram verdadeiros elefantíacos” (Pará, 1849, p.54-55). Assim, para o lazareto do Tucunduba também foram designadas pessoas acometidas por filariose, doenças mentais, varíola, febre amarela e epilepsia (Henrique, 2012, p.167). Com relação a 1848, o presidente da província informava que havia 68 internos no leprosário e que “os que no mapa se dão como curados, são enfermos de outras moléstias, cujas aparências induziram a errada classificação” (Pará, 1848, p.96). São essas questões que explicam a preocupação do presidente da província com o diagnóstico preciso dos leprosos que seriam submetidos à experiência de cura com o assacu.

O *Correio Mercantil*, da Bahia, publicou matéria revelando os nomes de quatro dos leprosos que haviam sido submetidos às experiências com o assacu pela Santa Casa de Misericórdia do Pará. Foram eles: “Antonio Hilário Martins, branco, solteiro, nascido em Monte Alegre”, que “tem o rosto inchado, fusco, rugoso e causa repugnância pela deformidade de suas feições”; Raymundo Gonçalves da Cunha, “branco, solteiro, nascido nesta cidade, filho de José da Cunha de Assunção”; “Domingos Manoel, preto, crioulo e escravo de João Henrique da Silva Lavareda, filho de Catarina Maria do Espírito Santo e de pai incógnito” e “Maria do Rosário, preta nascida na freguesia do Acará, escrava de Manoel Henrique Dias, filha de Michelle Francisca e de pai incógnito” (História..., 17 mar. 1848, p.1). Além dos nomes dos pacientes, os jornais costumavam publicar a descrição das doenças prévias dos doentes, o que constituía mais um meio de estigmatização que atingia a vida de populações pobres, fossem elas livres, libertas ou escravas.

O doutor José da Gama Malcher assim relatou os efeitos que o uso do assacu causou nos quatro leprosos: “logo depois que o tomaram sentiram abalo geral acompanhado de estremecimento ligeiro, sensação de frio nas extremidades, calor estendendo-se ao peito e ao rosto” (História..., 17 mar. 1848, p.1), “alguma ansiedade e vontade de vomitar” (p.2). Cada paciente vomitou entre 10 e 15 vezes. Alguns deles lançaram sangue pelo nariz, tiveram evacuações com sangue negro e sensação de que estavam sendo picados por formigas.^9^ O médico se mostrou esperançoso diante do quadro manifestado pelos doentes após o início do tratamento e parecia querer reivindicar para si todo o protagonismo da suposta cura:

Pelo que tenho expendido, parece-me que não serei leviano em esperar que, se progredirem as melhoras, os quatro infelizes poderão ficar habilitados para de novo pertencer à sociedade, de onde viviam proscritos. E, se tal consigo, que glória para a minha província! Quantos benefícios para a humanidade! E, que triunfo para a medicina! (História..., 17 mar. 1848, p.2).

Outros médicos procuraram ganhar notoriedade apresentando fórmulas específicas para o uso do assacu. Matéria dos *Anais de Medicina Brasiliense* informava que

o sr. cirurgião-mor Francisco de Paula Cavalcanti de Albuquerque (do Pará) acaba de comunicar a esta redação que, segundo um processo seu, tem ele preparado um extrato do leite de assacu, de que tem tirado grande vantagem, aplicando-o em seis doentes morféticos, que se estão tratando em seu lazareto particular (Extrato de assacu, jul. 1847, p.203).

Expressões como “se tal consigo” ou “segundo um processo seu”, referindo-se às experiências de médicos com o assacu, invisibilizavam o protagonismo do índio Antonio Vieira dos Passos.

Assim, a experiência de cura da lepra com o assacu ganhou repercussão nacional e “lazaretos particulares” surgiram em diversas partes do Brasil. Dizia uma matéria publicada no periódico *O Brasil*, do Rio de Janeiro: “Não chega do Pará barco que não traga notícias do assacu” (A morfeia!, 15 abr. 1848, p.3). O governo imperial solicitou à Imperial Academia de Medicina que fizesse experiências a fim de confirmar a eficácia do assacu para a cura da lepra (Brasil, 1848, p.21). Também no Rio de Janeiro, a Academia Médico-homeopática do Brasil ensaiou a cura da lepra com o assacu (Publicações..., 10 dez. 1847, p.2).

Houve, também, quem procurasse lucrar com a suposta cura da lepra. Matéria publicada no jornal *O Americano*, do Rio de Janeiro, dizia que “os genuínos e puros medicamentos do assacu para a cura da morfeia ou elefantíase acham-se tão somente em casa de Joaquim Bernardino Martins Caruncho, único agente no Império do Brasil. O anunciante apresentará documentos autênticos da eficácia destes medicamentos às pessoas que o exigirem” (Cura da morfeia, 29 abr. 1848, p.4).

As experiências com o assacu colocaram alguns personagens do Pará em evidência, especialmente porque muitos médicos paraenses enviaram cartas para pessoas e instituições de outras partes do Brasil, informando sobre a suposta cura da lepra. Os doutores Camillo do Valle, Gama Malcher e Joaquim Frutuoso Pereira Guimarães se tornaram sócios correspondentes da Academia Imperial de Medicina (Parecer..., out. 1847, p.279). Francisco da Silva Castro, juntamente com Guimarães, tornou-se colaborador dos *Anais de Medicina Brasiliense* (Correspondência particular, jul. 1847, p.204).

Quanto mais as experiências se espalhavam, mais ansiedade geravam em torno do veredito final da eficácia do assacu para a cura da lepra: “o assacu é como uma teia de aranha suspensa no vago das experiências terapêuticas no Rio de Janeiro. Aguardam-se as suas virtudes miraculosas contra a lepra, como se aguardou a verificação dos efeitos do guano contra essa moléstia e, todavia, o guano falhou” (Remédios novos, out. 1847, p.174). Outra matéria dizia: “Todos aguardam com impaciência o resultado das experiências feitas nos hospitais do Rio de Janeiro, Bahia e Pernambuco” (Plantas medicinais, out. 1847, p.218).

Tal foi a repercussão da experiência do índio Passos que ensaios de cura da lepra com o assacu foram feitos no Hospital de São Lázaro, em Lisboa, Portugal, com 13 doentes, concluindo os médicos que a substância poderia auxiliar na melhora, embora não tivesse poder de cura (Assacu, nov. 1848, p.123; Branco Jr., 1850).

Os desdobramentos da experiência do índio Antonio Vieira Passos mereceram registro do conceituado médico polonês Chernoviz, que obteve o título de doutor em medicina pela Escola Médica de Montpellier, França, sendo autor de manuais de medicina popular de ampla circulação no Brasil do século XIX (Guimarães, 2016). Foi ele quem escreveu, em 1841, o primeiro e mais importante dos manuais de terapêutica médica que circularam no Brasil do século XIX, o *Formulário e guia médico* (Chernoviz, 1996). Com a publicação, em 1842, do *Dicionário de medicina popular e das ciências acessórias,* tornou-se o médico mais conhecido no Brasil. Na quinta edição de seu dicionário, Chernoviz (1878, p.255) se refere ao assacu como “árvore do Pará”, cujo suco e cozimento da casca foram “recomendados no curativo da morfeia” e conta em detalhes o “método de tomar o remédio (que se usava no Pará)”. Registra, também, que na Europa foram feitas experiências com o assacu na cura da lepra. Dizia ele: “A humanidade acreditou por algum tempo no efeito do assacu contra a morfeia; porém as experiências feitas no Pará e nas demais províncias do Brasil, e na Europa, provaram que estas esperanças eram exageradas; e o assacu perdeu a reputação que tinha como remédio da morfeia” (p.255).

De fato, as experiências realizadas nos “lazaretos experimentais”, tanto no Brasil quanto na Europa, demonstraram que, nos primeiros meses do tratamento, o assacu propiciava melhora significativa nos sintomas da lepra, mas depois deixava de fazer efeito. O próprio José Joaquim de Souza Gomes, por exemplo, faleceu cerca de dois anos após ter aparecido “curado” em Santarém (Pará, 1849).

Dos 11 leprosos recolhidos no lazareto experimental de Belém, cinco faleceram, dois fugiram, três voltaram para o Hospital de Tucunduba e um se recolheu no Hospital da Caridade (Pará, 1848). Os efeitos do assacu sobre o organismo, conforme apresentados, ajudam a entender por que alguns leprosos fugiam do tratamento. Por outro lado, a recorrência do surgimento de supostas curas para a lepra fazia com que, vez ou outra, os doentes fossem submetidos a esse tipo de experiências e, todas, até então, haviam se revelado frustradas. Lembre-se que o próprio Souza Gomes havia fugido para Santarém, no interior da província do Pará, recusando-se a viver no leprosário do Tucunduba. Situação semelhante ocorria no Rio de Janeiro, conforme se vê na resposta do médico que tratou doentes com assacu no hospital dos lázaros dessa cidade: “Desde que recebi o assacu passei a empregá-lo em vários doentes deste hospital, mas até hoje só um deles, que tem sido mais assíduo e dócil em seguir o tratamento prescrito, oferece melhoras dignas de nota” (Hospital dos Lázaros, 29 abr. 1848, p.3). De fato, nem todos os doentes manifestavam disposição de agir como “corpos dóceis” (Foucault, 2009) diante das experiências a que os médicos os submetiam.

## Considerações finais

Decretado o insucesso do assacu na cura da lepra, não se falou mais no índio Antonio Vieira dos Passos, aquele que, reproduzindo experiências de seus ancestrais indígenas e sem ter sentado em um banco de escola de medicina, alimentou em pessoas do Brasil e do exterior a esperança de que nessa planta poderia residir o princípio eficaz para a cura da doença. Teria ele voltado para Santarém? Um documento existente no Cartório do 2º Ofício de Óbidos, no oeste do Pará, nos apresenta uma pista importante. Trata-se de um registro de óbito que dizia o seguinte:

No dia vinte de maio do ano mil oitocentos oitenta e três, em meu cartório compareceu Francisco Vieira dos Passos, morador no igarapé do Muratuba, lavrador, natural de Vila Franca, e declarou que nesse mesmo dia, às três horas da manhã, faleceu em sua casa onde morava no dito Muratuba sua mãe de nome Francisca (ilegível) da Conceição, idade setenta anos, casada com Antonio Vieira dos Passos, sem testamento, ficando de seu matrimônio cinco filhos (Óbitos, 1879-1888).

Se era o mesmo Antonio Vieira dos Passos, ele teria casado com Francisca da Conceição e tido cinco filhos, residindo no igarapé do Muratuba. Vila Franca, lugar de onde Francisco Vieira dos Passos era natural, é um lugar próximo a Santarém, onde o índio Passos foi preso.

A experiência do índio Passos evidencia que a busca pela cura da lepra não estava restrita ao campo da medicina oficial. Em diversas partes do mundo, curandeiros, pajés e tantos outros classificados como charlatães realizavam experiências de cura da doença. A propósito, não encontrei um único documento que classificasse Antonio Vieira dos Passos como charlatão, o que pode ser explicado pelo respeito que os médicos costumavam ter pela medicina indígena e pelo sucesso inicial da experiência com o assacu. Os curandeiros, em suas diversas modalidades, exerciam um ofício associado a um “trabalho manual, de baixo *status* social, reservado a grupos marginalizados da sociedade, como os escravos, os libertos, os pobres e as mulheres” (Guimarães, 2016, p.25). Eram desses estratos sociais as pessoas sobre as quais Antonio dos Passos dizia ter curado da lepra.

Aos leprosos, restava a estigmatização, a dor de ser retirado da vida em sociedade (especialmente quando pobres) e a submissão a todo tipo de experiências nos chamados “lazaretos experimentais”. Mas não apenas isso. Como vimos, alguns deles fugiam ou se recusavam a ser submetidos a essas experiências. E os que viviam nos leprosários não compactuavam com a visão desses locais como “cemitérios dos vivos” (Henrique, 2012).

Dada a imprecisão da fronteira entre a medicina oficial e as práticas de cura populares no século XIX, conforme demonstrado ao longo deste artigo, as maiores autoridades da província do Pará se dispuseram a conhecer o “método do índio Passos”, representante dos povos que, distante das figuras idealizadas do indianismo brasileiro, costumeiramente eram definidos nos relatórios dos presidentes da província como preguiçosos, símbolos do último grau de degeneração da espécie humana (Henrique, 2003). Se a experiência do índio Passos se revelou um fracasso para a cura da lepra, muitas outras realizadas por indígenas foram bem sucedidas e passaram a compor o conhecimento ocidental na cura de diversas doenças. Além da ipecacuanha (*Psychotria ipecacuanha* (Brot.) [Stokes]), utilizada em remédios contra tosse e xaropes para indução de vômito, da ayapana (*Ayapana triplinervis* [Vahl] R.M. King e H. Rob.) utilizada como tônico, digestivo e antidiarreico, cite-se o exemplo da quinina, substância extraída da casca da quina ou cinchona, descoberta pelos indígenas do Peru e utilizada na cura de febres. Pertencente ao gênero *Cinchona*, da família Rubiaceae, a quinina passou a ser utilizada pela medicina ocidental no tratamento da malária e, segundo Nieto Olarte (2006, p.19), é “a planta americana mais importante e controversa da história da medicina”. Outro exemplo é o da andiroba (*Carapa guianensis* [Aublet]), amplamente utilizada na Amazônia como analgésico, antibacteriano, anti-inflamatório, antifúngico, antialérgico, antimalárico, além de se mostrar eficaz contra feridas, hematomas, úlceras de herpes, reumatismo e infecções de ouvido (Sousa et al., 2019). Por sua vez, os médicos tratavam de, ao seu modo, aprimorar essas experiências, traduzindo-as em uma linguagem dita científica, de modo a torná-las mais legítimas e, quem sabe, eternizar seus próprios nomes como descobridores da cura da lepra.

As experiências que o índio Passos iniciou em Santarém, por volta de 1847, correram mundo afora, num circuito que, de certa forma, foi concluído com a decretação por Chernoviz de que a reputação do assacu como “remédio para morfeia” tinha chegado ao fim. Nesse caso, note-se que “um mundo examinado pela ‘lente’ da história da lepra mostra intricadas convergências de histórias nacionais, de políticas médicas, governamentais e internacionais” (Robertson, 2003, p.2). De todo modo, se a experiência com o assacu não teve início com o índio Antonio Vieira dos Passos, também não parou nele. Em 1921, o curandeiro colombiano Mamerto Cortés prometia, mais uma vez, a cura da lepra com o assacu, sendo definido como charlatão pelos médicos de Belém (Gomes, 2019).

Pesquisas sobre a história da medicina no Brasil do século XIX são fundamentais para recuperar o protagonismo indígena no processo de constituição e oficialização da medicina no país. Além de seu uso como mão de obra, os índios brasileiros tiveram papel de destaque na consolidação do conhecimento ocidental acerca de botânica, agronomia, medicina, entre outros campos do saber. Ao longo do século XIX, o saber indígena sobre o uso de plantas medicinais era amplamente reconhecido e utilizado pelos médicos com a intenção de incorporá-las em seu repertório terapêutico. O assacu foi abandonado como tratamento para a lepra, mas continuou sendo usado com outras finalidades. Em 1932, Paul Le Cointe (1947, p.55-56), por exemplo, dizia que “não tem fundamento a reputação que tem essa seiva de curar a morfeia”, mas “a infusão das flores masculinas (espigas) ou as brácteas frescas, aplicam-se nos furúnculos; o efeito é muito rápido e deve ser interrompido logo que o furúnculo começa a amolecer. As folhas trituradas com água aplicam-se nos reumatismos”.

Se a experiência com o assacu não teve como resultado a esperada cura da lepra, o uso medicinal que os índios faziam de muitas outras plantas foi incorporado pela medicina oficial, a exemplo da ipecacuanha, da ayapana, da copaíba (*Copaifera langsdorffii* Desf.) e tantas outras. Recuperar essas experiências é, também, um modo de registrar o protagonismo indígena na história do Brasil e na história da medicina.

## References

[B1] A MORFEIA! *O Brasil*, p.3, 15 abr. 1848.

[B2] ALMEIDA, Mauro Barbosa de; CUNHA, Manuela. *Enciclopédia da floresta*. São Paulo: Companhia das Letras. 2002.

[B3] ASSACU. *Anais de Medicina Brasiliense*, v.4, n.5, p.123, nov. 1848.

[B4] BAENA, Antônio Ladislau Monteiro. *Ensaio corográfico sobre a província do Pará*. Brasília: Conselho Editorial/Senado Federal, 2004.

[B5] BELTRÃO, Jane Felipe. *Cólera, o flagelo da Belém do Grão-Pará*. Belém: Universidade Federal do Pará, 2004.

[B6] BENCHIMOL, Jaime Larry; SÁ, Magali Romero. Adolpho Lutz and controversies over the transmission of leprosy by mosquitoes. *História, Ciências, Saúde – Manguinhos*, v.10, supl.1, p.49-93, 2003. 14650407

[B7] BRANCO JR., José Maria Alves. Ensaios com o assacu no tratamento da morfeia no Hospital de S. Lazaro. *Jornal da Sociedade Pharmaceutica Lusitana, t.1*. Lisboa: Na Imprensa Silviana, 1850. p.233-239.

[B8] BRASIL. Lei n.9.010, de 29 de março de 1995. Dispõe sobre a terminologia oficial relativa à hanseníase e dá outras providências. Disponível em: http://www.planalto.gov.br/ccivil_03/Leis/L9010.htm. Acesso em: 20 jun. 2011. 29 mar. 1995.

[B9] BRASIL. Relatório da Repartição dos Negócios do Império apresentado à Assembleia Geral Legislativa na 1ª Sessão da 7ª Legislatura pelo respectivo ministro e secretário de Estado visconde de Macaé. Rio de Janeiro: Tipografia Nacional, 1848.

[B10] CABRAL, Dilma Fátima Avellar. *Entre ideias e ações: medicina, lepra e políticas públicas de saúde no Brasil (1894-1934)*. Tese (Doutorado em História) – Universidade Federal Fluminense, Niterói, 2007.

[B11] CERTEAU, Michel de. A beleza do morto. In: Certeau, Michel de. *A cultura no plural*. Campinas: Papirus, 1995.

[B12] CHERNOVIZ, Pedro Luiz Napoleão. *A grande farmacopeia brasileira: formulário e guia médico: uma guia das plantas medicinais brasileiras*. Belo Horizonte: Itatiaia, 1996.

[B13] CHERNOVIZ, Pedro Luiz Napoleão. *Dicionário de medicina popular e das ciências acessórias*. v.1, A-F. Paris: Em Casa do Autor, 1878.

[B14] CHIARADIA, Clóvis. *Dicionário de palavras brasileiras de origem indígena*. São Paulo: Limiar, 2008.

[B15] CONSTA-NOS QUE O SR..., *Gazeta Oficial*, p.2, 4 set. 1859.

[B16] CORRESPONDÊNCIA PARTICULAR. *Anais de Medicina Brasiliense*, v.3, n.2, p.204, jul. 1847.

[B17] CURA DA LEPRA. *Publicador Maranhense*, p.3, 9 mar. 1844.

[B18] CURA DA MORFEIA. *O Americano*, p.4, 29 abr. 1848.

[B19] CURA DA MORFEIA. *Diário do Rio de Janeiro*, p.3, 5 ago. 1847.

[B20] DUARTE, Regina Horta. *História e natureza*. Belo Horizonte: Autêntica, 2005.

[B21] ELEFANTÍASIS. *Treze de Maio*, p.71, 4 jul. 1840.

[B22] EXTRATO DE ASSACU. *Anais de Medicina Brasiliense*, v.3, n.2, p.203, jul. 1847.

[B23] EXTRATO DA VIAGEM ao Brasil pelo reverendo padre Daniel Kidder. *Gazeta Oficial do Império do Brasil*, p.3, 25 fev. 1848.

[B24] FIGUEIREDO, Aldrin Moura de. *A cidade dos encantados: pajelanças, feitiçarias e religiões afro-brasileiras na Amazônia, 1870-1950*. Belém: EdUFPA, 2008a.

[B25] FIGUEIREDO, Aldrin Moura de. Assim como eram os gafanhotos: pajelança e confrontos culturais na Amazônia do início do século XX*.* In: Maués, Raymundo Heraldo; Villacorta, Gisela Macambira (org.). *Pajelanças e religiões africanas na Amazônia*. Belém: EdUFPA, 2008b. p.53-102.

[B26] FIGUEIREDO, Aldrin Moura de. Pajés, médicos e alquimistas: uma discussão em torno de ciência e magia no Pará oitocentista. *Cadernos do Centro de Filosofia e Ciências Humanas*, v.12, n.1-2, p.41-54, 1993.

[B27] FOUCAULT, Michel. *Vigiar e punir: nascimento da prisão*. Petrópolis: Vozes, 2009.

[B28] FREYRE, Gilberto. *Casa-grande e senzala*. Rio de Janeiro: José Olympio, 1946.

[B29] GOFFMAN, Erving. Estigma: notas sobre a manipulação da identidade deteriorada. Rio de Janeiro: Zahar, 1975.

[B30] GOMES, Elane Cristina Rodrigues. *A lepra e a letra: escrita e poder sobre a doença na cidade de Belém (1897-1924)*. Tese (Doutorado em História) – Universidade Federal do Ceará, Fortaleza, 2019.

[B31] GOODY, Jack. *A domesticação da mente selvagem*. Petrópolis: Vozes, 2012.

[B32] GUIMARÃES, Maria Regina Cotrim. Civilizando as artes de curar: Chernoviz e os manuais de medicina popular do Império. Rio de Janeiro: Editora Fiocruz, 2016.

[B33] HARRIS, Mark. “What it means to be ‘caboclo’”: some critical notes on the construction of Amazonian ‘caboclo’ society as an anthropological object. *Critique of Anthropology*, v.18, n.1, p.83-95, 1998.

[B34] HENRIQUE, Márcio Couto. Sem Vieira nem Pombal: índios na Amazônia do século XIX. Rio de Janeiro: EdUerj, 2018.

[B35] HENRIQUE, Márcio Couto. Escravos no purgatório: o leprosário do Tucunduba (Pará, século XIX). *História, Ciências, Saúde – Manguinhos*, v.19, supl., p.153-177, 2012. 10.1590/s0104-59702012000500009 23370104

[B36] HENRIQUE, Márcio Couto. *O general e os tapuios: linguagem, raça e mestiçagem em Couto de Magalhães (1864-1876)*. Dissertação (Mestrado em Ciências Sociais) – Universidade Federal do Pará, Belém, 2003.

[B37] HISTÓRIA de quatro morféticos que têm tomado o assacu. *Correio Mercantil,* p.1, 17 mar. 1848.

[B38] HOSPITAL DOS LÁZAROS. *Correio Mercantil e Instrutivo, Político, Universal*, p.3, 29 abr. 1848.

[B39] INSTRUÇÕES a que se refere o ofício dessa data. *Gazeta Oficial*, p.1, 30 jun. 1859.

[B40] INTERIOR. *Publicador Maranhense*, p.3, 22 fev. 1848.

[B41] LE COINTE, Paul. *Amazônia brasileira III: árvores e plantas úteis (indígenas e aclimatadas).* São Paulo: Companhia Editora Nacional, 1947.

[B42] MARQUES, Vera Regina Beltrão. *Remédios secretos, saberes e poderes*. In: Congresso Internacional de Americanistas, 49., 1997, Quito. *Anais...*, Quito: Pontificia Universidad Católica del Equador, 1997. Disponível em: http://www.naya.org.ar/congresos/contenido/49CAI/Marques.htm. Acesso em: 6 jul. 2011.

[B43] MARRAS, Stelio. A propósito de águas virtuosas: formação e ocorrências de uma estação balneária no Brasil. Belo Horizonte: Editora UFMG, 2004.

[B44] MARTINS, Patrícia Vieira; CAPONI, Sandra. Hanseníase, exclusão e preconceito: histórias de vida de mulheres em Santa Catarina. *Ciência e Saúde Coletiva*, v.15, supl.1, p.1047-1054, 2010. 10.1590/s1413-81232010000700011 20640261

[B45] MARTIUS, Karl Friedrich Philipp von. Natureza, doenças, medicina e remédios dos índios brasileiros. São Paulo: Nacional, 1979.

[B46] MARTIUS, Karl Friedrich Philipp von. *Sistema de matéria médica vegetal brasileira*. Rio de Janeiro: Eduardo e Henrique Laemmert, 1854.

[B47] MARTIUS, Carlos Frederico Ph. Como se deve escrever a História do Brasil. *Revista do Instituto Histórico e Geográfico Brasileiro*, n.6, p.389-411, 1844.

[B48] MONTEIRO, Yara Nogueira. Violência e profilaxia: os preventórios paulistas para filhos de portadores de hanseníase. *Saúde e Sociedade*, v.7, n.1, p.3-26, 1998.

[B49] MORAES, Alexandre José de Mello. *Fitografia ou Botânica brasileira aplicada à medicina, às artes e à indústria, seguida de um suplemento de matéria médica, inclusive as plantas conhecidas e aplicadas pelos índios em suas enfermidades*. Rio de Janeiro: Livraria de B.L. Garnier, 1881.

[B50] NIETO OLARTE, Mauricio. Remedios para el imperio: historia natural y la apropiación del Nuevo Mundo. Bogotá: Universidad de los Andes, 2006.

[B51] ÓBITOS. 31 nov. 1879 a 5 out. 1888, n.1 a n.596, n.144. (Cartório do 2º Ofício de Óbidos, Óbidos, PA). 1879-1888.

[B52] OFÍCIOS da Santa Casa de Misericórdia do Pará. Fundo Secretaria da Presidência da Província; série 13; caixa 78 (Arquivo Público do Estado do Pará, Belém). 13 abr. 1849.

[B53] OFÍCIOS da Santa Casa de Misericórdia do Pará. Fundo Secretaria da Presidência da Província; série 13; caixa 78 (Arquivo Público do Estado do Pará, Belém). 20 dez. 1848.

[B54] OS MÉDICOS DO PARÁ... *Iris: Periódico de Religião, Belas Artes, Ciências, Letras, História, Poesia, Romance, Notícias e Variedades*, t.1, n.1, p.125-126, 1848.

[B55] PARÁ. Fala dirigida pelo Exmo. Sr. Conselheiro Jerônimo Francisco Coelho, presidente da Província do Grão-Pará à Assembleia Legislativa Provincial na abertura da segunda sessão ordinária da sexta legislatura, no dia 1^o^ de outubro de 1849. Pará: Tipografia de Santos e Filhos, 1849.

[B56] PARÁ. Fala dirigida pelo Exmo. Sr. Conselheiro Jerônimo Francisco Coelho, presidente da Província do Grão-Pará, à Assembleia Legislativa Provincial, na abertura da sessão ordinária da sexta legislatura, no dia 1^o^ de outubro de 1848. Pará: Tipografia de Santos e Filhos, 1848.

[B57] PARÁ. Discurso recitado pelo Exmo. Sr. Dr. João Maria de Moraes, vice-presidente da Província do Pará na abertura da segunda sessão da quinta legislatura da Assembleia Provincial no dia 15 de agosto de 1847. Pará: Tipografia de Santos e Filhos, 1847.

[B58] PARECER sobre o assacu apresentado e aprovado pela Academia de Ciências Médicas da Bahia pelo dr. M. A. de M. Albuquerque Pitta. *Arquivo Médico Brasileiro*, t.4, n.1, p.274, out. 1847.

[B59] PLANTAS MEDICINAIS. *Arquivo Médico Brasileiro*, t.4, n.1, p.218, out. 1847.

[B60] PROJETO. *Treze de Maio*, p.146, 2 out. 1840.

[B61] PUBLICAÇÕES a pedido. *Jornal do Commercio*, p.2, 10 dez. 1847.

[B62] REMÉDIOS NOVOS. *Arquivo Médico Brasileiro*, t.4, n.1, p.174, out. 1847.

[B63] REMÉDIOS vulgares do Pará. *Arquivo Médico Brasileiro*, t.3, n.11, p.244, jul. 1847.

[B64] ROBERTSON, Jo. Editor’s note. *História, Ciências, Saúde – Manguinhos*, v.10, supl.1, p.1-4, 2003. 10.1590/s0104-59702003000400002 14650405

[B65] ROMERO-SALAZAR, Alexis et al. El estigma en la representación social de la lepra. *Cadernos de Saúde Pública*, v.11, n.4, p.535-542, 1995. 10.1590/s0102-311x1995000400002 12973588

[B66] SAMPAIO, Gabriela. Nas trincheiras da cura: as diferentes medicinas no Rio de Janeiro imperial. Campinas: Unicamp, 2001.

[B67] SANJAD, Nelson; COSTA, Ejhon. Medicina e circulação de saberes no Grão-Pará do século XIX: os experimentos terapêuticos de Antônio Corrêa de Lacerda (1777-1852) e Francisco da Silva Castro. In: Franco, Sebastião Pimentel et al. *Uma história brasileira das doenças*. Belo Horizonte: Fino Traço, 2019. p.39-62.

[B68] SANJAD, Nelson; PATACA, Ermelinda; SANTOS, Rafael Rogério Nascimento dos. Knowledge and circulation of plants: unveiling the participation of Amazonian indigenous peoples in the construction of eighteenth and nineteenth century botany. *HoST: Journal of History of Science and Technology*, v.15, n.1, p.11-38, 2021.

[B69] SANTOS FILHO, Lycurgo de Castro. *História Geral da Medicina Brasileira, v.1*. São Paulo: Hucitec, 1977.

[B70] SAÚDE PÚBLICA. *A Revista*, p.5, 18 ago. 1848.

[B71] SIGAUD, J.F. Xavier. *Do clima e das doenças do Brasil ou estatística médica deste Império*. Rio de Janeiro: Editora Fiocruz, 2009.

[B72] SOUSA, Ronaldo Lopes de et al. Extração e comercialização do óleo de andiroba (“Carapa guianensis” Aublet) na comunidade da ilha das Onças, no município de Barcarena, Pará, Brasil. *Interações*, v.20, n.3, p.879-889, 2019. Disponível em: 10.20435/inter.v0i0.1826. Acesso em: 5 maio 2022.

[B73] TENDO CHEGADO... *Diário Novo*, p.2, 16 out. 1847.

[B74] VIEIRA, Erika Fernanda de Matos et al. Mururé (“Brosimum acutifolium” Huber) in the treatment of syphilis in colonial Amazonia: historical data to the actual contribution to treatment. *Acta Botanica Brasilica*, v.33, n.2, p.183-190, abr.-jun. 2019. Disponível em: 10.1590/0102-33062019abb0030. Acesso em: 14 dez. 2021.

[B75] VILA DE SANTARÉM, 16 de julho de 1847. *Jornal do Commercio*, p.2, 21 set. 1847.

