# Supplementary material for: Do índio Passos ao doutor Chernoviz: experiências de cura da lepra no Pará do século XIX
Source: Hist Cienc Saude Manguinhos. 2023 Aug 4;30:e2023027. [Article in Portuguese] doi: 10.1590/S0104-59702023000100027 (PMC10402912; doi:10.1590/S0104-59702023000100027)
Supplement: Translation

# Translation: From Passos the Indian to Doctor Chernoviz: experiments to cure leprosy in nineteenth-century Pará

**DOI:** 10.1590/S0104-59702023000100027en

**Authors:** Márcio Couto Henrique

**Affiliations:** i Brazil Professor, Faculdade de História/Universidade Federal do Pará. Belém – PA – Brazil. orcid.org/0000-0002-0170-5315. marciocouto@ufpa.br

**Keywords:** Leprosy, Medicine, Indigenous protagonism, Amazon

## Abstract

This article analyzes an experiment to cure leprosy using the assacu plant (*Hura crepitans* L.) conducted in Santarém, Pará, in 1847, by an Indigenous man named Antonio Vieira dos Passos. The experiment was later repeated in other Brazilian provinces and abroad. This article establishes relationships between medical practices in other parts of the country while focusing on the dialog between official and Indigenous medicine. Newspaper articles and official documents of the time show that Indigenous knowledge of medicinal plants was widely recognized and utilized by physicians wishing to incorporate it into the official therapeutic repertoire.

## The beauty of the dead

When he saw his skin covered with lumps, his fingers atrophied, and his face deformed, 50-year-old Marianno José Machado went to live in the Lepers’ Hospital in Rio de Janeiro. The stigma (Goffman, 1975)^1^ that accompanied the disease then known as leprosy made daily life in society impossible.^2^ Marianno was so miserable he decided to undergo a risky experiment that some said could potentially free him of his terrible disease: allowing himself to be bitten by a rattlesnake. After signing a statement that he assumed responsibility for his actions, he put his right hand into the cage that housed the serpent, which immediately bit his finger.

A report ten years afterward reported that Marianno decided to subject himself to this experiment “despite the prudent counsel of many physicians who doubted whether this dangerous method could be successful”^3^ (Extrato da viagem..., 25 fev. 1848, p.3). In fact, no one attempted to stop the experiment, and Marianno “suffered cruelly until midnight.” The few medications administered by the doctors present did nothing to alleviate his suffering, and the report reveals the passive, observational stance of those who watched from the snakebite at 11:50 in the morning of September 4, 1838 until 11:30 the next morning, when Marianno “gave up his soul to God.” Driven by scientific curiosity and indifferent to the patient’s suffering, the observers made a minute-by-minute report of his reactions, indicating that he was encouraged to share everything he felt: the blood gushing from the bite wound, the swelling in his hand, chills, vision changes, tingling face, anxiety, throat swelling, chest pain, irregular heartbeat, until the moment he died (Extrato da viagem..., 25 fev. 1848, p.3). In 1844, the French Doctor Xavier Sigaud (2009, p.271) recorded the final frustration among the physicians a day after Marianno’s death: “The stench emerging at 10:00 in the morning of the following day is enough to impede the autopsy, to the great disappointment of those [who practice] this art.”

Less tragic experiments to cure leprosy were conducted throughout Brazil during the nineteenth century, and contemporary newspapers often announced supposed cures for the disease, in Brazil as well as abroad. A cure for the “horrible disease of leprosy” discovered by a Portuguese woman in the city of Porto was announced in 1844 (Cura da lepra, 9 mar. 1844, p.3). In 1848, the discovery of a “radical cure for leprosy using herbs and other items” was reported in the village of Itapetininga, São Paulo (Saúde pública, 18 ago. 1848, p.5). This discovery was made by a Frenchman named Charles Pierre Etéchéion, who since 1847 had experimented with cures for leprosy that involved guano, the feces of birds and bats (Cabral, 2007). In 1859, in the small village of Paracari, near Santarém, Pará, the healer Antonio Francisco da Costa declared he could cure leprosy with a treatment he named after the village (Instruções..., 30 jun. 1859, p.1).

An 1840 article in the *Treze de Maio* newspaper depicts public perception of the disease at that time, growing numbers of leprosy cases in the province of Pará, and the hope that such reporting generated:

No one is unfamiliar with the terrible and progressive scourge that day by day becomes a greater threat to our province: leprosy! We all feel the urgent need to take more serious measures and cautions so that this same evil does not spread throughout the population, become widespread, and ultimately we will only have our own apathy to blame (Projeto, 2 out. 1840, p.146).

Leprosy was consequently seen as a “terrible scourge,” a threat to society, an evil to be driven out of the province or at least brought under control. To do this, the author maintained that the provincial government should build a leper’s hospital in some far-off place and take measures to send lepers to Goiás Province “in order to use the Caldas” (Projeto, 2 out. 1840, p.146). The hot springs at Caldas Novas, in Goiás, as well as those at Poços de Caldas in southern Minas Gerais, were considered effective aids in curing diseases like leprosy, syphilis, and venereal diseases in general. Many people who were disillusioned with the ordinary resources they could obtain in the villages and cities placed their hopes in the healing power of these waters (Marras, 2004). The disease was known by other names in the past in Portuguese, such as *morfeia*, *gafa*, *elefantíase dos gregos*, *elefancia*, *mal de lázaro*, *mal da pele*, and *mal do sangue*. It was synonymous with everything considered repugnant that could corrupt morality, destroy social life, and topple the natural order of things.

In June 1847, readers of the *Treze de Maio* newspaper in Belém, Pará learned that a cure for leprosy had been found.^4^ In investigating this event, provincial vice-president João Maria de Moraes learned that a certain José Joaquim de Souza Gomes, long known to have leprosy, appeared to be cured of the disease after applications of assacu (*Hura crepitans* L.) by an Indian named Antonio Vieira dos Passos (Pará, 1847, p.19). The Polish physician Chernoviz described the assacu tree in 1842 as follows:

A colossal tree, with serrated oval subchordate leaves; fruit, with woody capsule, multilocular, with one seed in each locule. Through incision, a sticky whitish-tan or reddish white sap is extracted from this tree, which condenses and solidifies with difficulty as it runs; when condensed it is dark tan, looking more like gum than resin, and is very soluble in water (Chernoviz, 1878, p.254-255).

Prior to that time, native peoples were known to have used assacu, as described by Martius (1854, p.167): “The milky sap of this equatorial tree is utilized by the Indians as an anthelminthic and to stun fish” so they could be caught with bare hands.

At the time, Passos was in jail in Santarém, accused of murder. The Santarém city council immediately decided to conduct experiments with assacu on five people with leprosy, overseen by the surgeon who practiced in the village, Raymundo José Rebello. Rebello summoned Passos and interrogated him in Santarém, describing him as “having a resourceful and energetic spirit, though rustic, reads little to nothing, but can sign his name” (Vila de Santarém..., 21 set. 1847, p.2). Passos was not intimidated:

He regularly states that he is not afraid to cure any leper while supervised by physicians. But he does not hesitate to say: whoever has to die of this disease always dies, perhaps meaning that just as other diseases with known remedies are not always cured by them, his cure for leprosy is also not infallible. He says he has not declared all the remedies he applies. But it seems that the surgeon Rebello has all of them, or [at least] the essential ones (Vila de Santarém..., 21 set. 1847, p.2).

This report is important, since it reveals (albeit indirectly) Passos’s secure defense of the method he used to cure leprosy and the comparative reading he made between his healing practice and official medicine, both fallible or unable to cure every single case. The report also suggests that Passos took pains to maintain a certain control over the medications he used for himself, not reporting all of them. Four months after the experiment in Santarém began, the surgeon reported that the patients had noticeably improved and there was hope they might be cured after another three months of treatment.

Various newspapers published the recipe used by Passos:

Mr. Rebello said that the way he uses assacu to cure lepers in Santarém is what was used by Souza Gomes to treat this disease and that he only perfected and adapted it to the rules of Medical Science. It is based on daily use of pills made of the condensed sap (referred to as the extract); on using a mixture composed of half a pound of strong cooked bark and ten to twenty drops of the juice (known as the emetic); the patient must drink the entire mixture all at once to make himself vomit, and patients consequently vomit more than six times. This is repeated daily for eight days. The use of overall baths prepared by the saturated cooking of the bark, repeated for days, and the use of a cooked emollient for everyday drinking (Interior, 22 fev. 1848, p.3).

Note that Rebello, the surgeon, tried to differentiate his procedure from the one used by Passos, stating that he “perfected” and “adapted” the method “to the rules of Medical Science.” This scientific clothing was intended to lend this experimental treatment greater credibility and legitimacy.

An article published in the *Diário do Rio de Janeiro* newspaper reported receiving a letter from Santarém stating that “it appears that a poison named assacu produced in Pará, when applied as an emetic and purgative as taught by a Parintins Indian, completely cures leprosy” (Cura da morfeia, 5 ago. 1847, p.3). This was the only publication on this case with a reference to the potential ethnic background of Antonio Vieira dos Passos. In the seventeenth century, Jesuit missionaries founded a mission in Tupinambaranas, far from the current city of Parintins. In 1803, Maué and Munduruku Indigenous peoples were brought together there. Parintins became its official name in 1880, a reference to the Parintintins who had lived in the region. In 1832, Baena (2004, p.340; originally published in 1839) indicated that 970 Indians lived in the village, which at that time was called Vila Nova da Rainha. So the detail that the medication to cure leprosy was discovered by “a Parintins Indian” is a generic reference, and Antonio Vieira dos Passos may have been a member of any of the Indigenous peoples who lived in that region. In any case, it is important to call attention to this information, since the ethnic identity and even names of the Indians who provided information or revealed plants with medicinal uses to “scientific” medicine are traditionally erased. One example involves an herb known as ayapana (*Ayapana triplinervis* (M. Vahl) R.M. King & H. Rob.) revealed by an Indigenous woman of unknown name and ethnicity which nevertheless became known as *erva-do-ouvidor* [ombudsman’s herb] in a reference to a bureaucrat in Belém known only for sending this plant to Lisbon and recommending its therapeutic properties (Sanjad, Pataca, Santos, 2021). Ayapana is an effective treatment for gastrointestinal disorders, mouth ailments, fever, and parasites, and is also used as a diaphoretic and antidote for snakebite.

Throughout the nineteenth century, physicians and scholars were especially interested in the remedies employed by the Indians. The voyages Karl F.P. von Martius undertook in Brazil between 1817 and 1820 resulted in a number of works including *Natureza, doenças, medicina e remédios dos índios brasileiros* [*Nature, disease, medicine, and remedies of the Brazilian Indians*] (Martius, 1979), originally published in 1844. In a chapter entitled “Medications from the plant kingdom,” Martius (1979, p.152) states that “these medicinal plants also have, in the fresh state in which they are employed by savage medicine, more effective medicinal value and in many cases fortunately and successfully replace the chemical compounds of European medicine.” Despite considering that the knowledge of “savage medicine” was “an expression of the grossest empiricism,” he did state that this topic “deserves all the attention of rational medicine.” So while Martius considered a hierarchy of knowledge, denying the Indians rational knowledge of the healing effects of the medicinal plants they utilized,^5^ he also believed that this same knowledge merited attention from “rational” science. This perception is certainly related to the image Martius (1844) had of Brazil’s Indians, which he saw as degenerated peoples who would soon disappear. Despite skepticism, Martius (1979, p.152) reported witnessing healing by “Indigenous doctors” on various occasions, in some cases so quickly and effectively that they “struck with rays of wonder,” as if the “mythical plants” used by the Indians not only cured but performed miracles. In fact, the author stated that because it was impossible to confirm that they were discovered by the Indians, and because they generally had origin myths surrounding them, “with regard to these plants we should admit they came from Paradise” (p.155).

An article published in Rio de Janeiro about assacu reveals this mixture of discredit and curiosity around Indigenous medicine:

Doctors in Pará are talking about a very important discovery for science resulting from the practices of the Indians. They say that leprosy can be excellently cured with assacu, a plant in the family Eforbiacea named *Ura brasiliensis* by Dr. Martius. A similar virtue and antisyphilitic [properties] are attributed to various other plants from Pará [such as] manacá, mururé, cururu-timbó etc.

... There are those, however, who doubt this proclaimed virtue, and remembering the fate of guano in an identical case, we await the results of new experiments without forgetting that the positive effects of various *ipecacuanhas* and other plants were taught to us by the Indians (Os médicos do Pará..., 1848, p.125-126).

Note that the author attributes the discovery to “the practices of the Indians,” just as Martius attributed Indigenous knowledge to the “grossest empiricism.” And to those who questioned the “proclaimed virtue” of the Indians in discovering medicinal plants, the author recalls the example of ipecac and other plants whose healing uses “were taught to us by the Indians.”^6^ Thus the paradox of a group of people considered degenerates, on their way to extinction, but from whom western science can obtain important contributions before this “natural but deteriorating knowledge” disappears forever. It was consequently necessary to take advantage of the Indians’ “natural knowledge,” considered remnants of a glorious era in a mythical past, of which nineteenth-century Indians were but a pale representation. The article consequently urged readers to appropriate the “beauty of the dead” (Certeau, 1995), translating Indigenous knowledge into a scientific language that would neutralize its supposed dangers. The effects of assacu needed to be scientifically proven with tests, experiments, evidence and counterevidence, otherwise this knowledge would be classified as merely empirical and not scientific, incapable of proving the plant’s pharmacological efficacy.

According to a publication in the *Arquivo Médico Brasileiro*, “there is no doubt that Pará, for its love of luxury and the magnificence of its vegetation, provides a series of health-giving plants for all physical ills... It is no wonder, then, that one reads in the recent issues of the newspapers of this province about leprosy cured by the poison assacu, and pulmonary arthritis [cured] with *mata-matá* bark” (Remédios..., jul. 1847, p.244). By highlighting the “magnificence of its vegetation,” the writer reinforces the idea of some “natural wisdom” held by the Indians, who themselves are seen as part of nature, according to the notion of the “ecological Indian” (Duarte, 2005). In any case, articles like these from Pará drew the attention of physicians. Sigaud (2009, p.131) stated that Pará “is the richest of all Brazil’s provinces in food plants and spices.” In 1859, Francisco da Silva Castro, one of the most renowned physicians in Belém, gathered other doctors in his home “so they could hear a reading of a work of his own authorship entitled *Enumeração dos vegetais indígenas do Brasil empregados em Medicina e mais usados, sua ação, doses, fórmulas etc.* [List of Brazilian indigenous plants used in medicine and the most common, their actions, doses, formulas etc.],” which was also part of a *materia medica* compendium pertaining to a Mr. Beirão, a Portuguese physician (Consta-nos que o Sr..., 4 set. 1859, p.2).

In this way, we can understand the doctors’ curiosity about the discovery of the Indian Passos’s supposed cure for leprosy. In a statement sent to the Pará provincial president Herculano Ferreira Pena, a Santarém district court judge named João Baptista Gonçalves Campos described his encounter with “the *pardo*^7^ Antonio Vieira dos Passos,” who confirmed he had cured José Joaquim de Souza Gomes:

He further declared that he was able to completely cure a Tapuyo [Indian] named Theodózio, a member of the Faro work gang who had been miserably wounded by leprosy but today carries out his work clearing brush in that village. [And] that he learned this cure from an old *curibocolo* in Juruti Parish named Manoel Joaquim, who was also in the work gang, but has since died (Vila de Santarém..., 21 set. 1847, p.2).

The detail that Antonio Vieira dos Passos had learned his cure from “an old *curibocolo* in Juriti Parish” reveals that the use of assacu to treat leprosy went back even farther among the Indians. *Curibocolo* is a corruption of *curiboca* or *caraiboca*, a term which according to the *Dicionário de palavras brasileiras de origem indígena* means *caboclo*, “an Indian/Black child” or “white/Indian mix” (Chiaradia, 2008, p.166). Years later, Mello Moraes (1881, p.58-59) said that Antonio Vieira dos Passos “used assacu milk for this cure, and that a *caboclo* from Juruti, Pará, named Manoel José Joaquim had taught him this medicine.” In nineteenth-century Amazonia, after they were baptized and learned rudimentary Portuguese, the Indians’ ethnic identity was negated, and they were called *caboclos* or *tapuyos* (Harris, 1998; Henrique, 2018). Baena (2004, p.235) states that in 1832, 385 Munduruku and Maué people lived in Juruti. It is consequently clear that assacu had been used by the native peoples of this region in western Pará to cure leprosy in places like Parintins and Juruti. On the other hand, it is clear that far from the medical knowledge of the cities, people like the “Tapuyo Theodózio” in Faro or Eusébia, the black woman in Vila Franca, “who is also cured of this same illness” (Tendo chegado..., 16 out. 1847, p.2), went to native healers to treat their diseases.

In 1840, president of Pará João Antonio de Miranda invited a group of physicians to evaluate a group of people presented as healers of leprosy by the Armada’s surgeon, Marcelo Domingues Barbosa. Doctors Camillo José do Valle Guimarães, Francisco da Silva Castro, José Custódio da Fonseca Paes, and Alexandre da Costa Araújo moved to disqualify the healer, considering him a quack and saying that some of the people he claimed to have cured of leprosy in fact never had the disease. They also claimed that he used known medical remedies in a peculiar manner, together with “extravagant compounds” that included plants known as mururé (*Brosimum acutifolium* Huber),^8^ marapuama (*Ptychopetalum olacoides* Bentham), and assacu (Elefantíasis, 4 jul. 1840, p.72). This illustrates that Passos was not the first healer to use assacu to cure leprosy.

According to Gilberto Freyre (1946, p.598), “doctors and healers were never very far from each other prior to the second half of the nineteenth century.” Plants that were unknown or had not been studied in detail by official medicine were widely used in experiments to cure a wide array of diseases. As for the therapeutic practices used by Antônio Corrêa de Lacerda and Francisco da Silva Castro, Sanjad and Costa (2019, p.57) note that “an openness to experiments based on popular and Indigenous knowledge can be observed, particularly the use of medicinal plants. This seems to have been common among the doctors who worked in the Amazon region before the emergence of bacteriology and so-called scientific medicine.” In this way, physicians replicated the conduct they tended to criticize among the Indians, using an entirely experimental and empirical pharmacological arsenal rooted in local uses and customs. Within this context, the line separating medicine from other healing practices was not yet well defined, leading doctors in Brazil’s provinces to experiment widely with popular cures, as indicated by ethnobotanical inventories that recorded any and all knowledge that could potentially be applied in medicine and industry (Sampaio, 2001; Guimarães, 2016).

## From the village out into the world

In October 1847, José de Souza Gomes (who lived in Santarém and claimed to have been cured of leprosy by the Indian Antonio Vieira Passos) traveled to Belém, where he was examined at the Governor’s Palace by a medical board comprised of Doctors Camillo José do Valle Guimarães, José da Gama Malcher, and Joaquim Frutuoso Guimarães. Their report to the president of the province contains some details that give us an idea of his background. The doctors stated that José de Souza Gomes was the son of Antonio de Souza Gomes, had been born in Belém, Pará, and was 33 years old. He was single, “mixed race, with a lymphatic temperament, with the constitution related to this temperament, had had an unrestrained lifestyle; had boils and syphilitic disease” (Interior, 22 fev. 1848, p.2). Three years earlier, he had been hospitalized in Belém at the Charity Hospital to treat a skin disease he did not know was leprosy, and was directed by the physician José da Gama Malcher to enter the leper asylum in Tucunduba. Displeased with this directive, Gomes fled the hospital to the interior of the province “in search of death,” according to the doctors. Then,

there an individual suggested he could cure his disease using assacu; the patient, miserable because of his awful state, accepted this offer of a cure, but doubted what was promised, and hoped that this method, like a poison, would shorten his days, a belief that soothed him in the face of his troubles; but this was not what happened, in fact this remedy affected his illness such that he was able to return to society, from which he had been cut off (Interior, 22 fev. 1848, p.2).

Next, the doctors discussed Gomes’s condition when he was first examined by Malcher at the Charity Hospital, before he had fled to the interior. The doctors described symptoms typically seen in people with leprosy, such as foul breath, raspy voice, a swollen, dull, wrinkled face that “was repugnant because of its deformed features,” swollen legs and deformed fingers (Interior, 22 fev. 1848, p.2). This description of leprosy symptoms helped reinforce the stigma around this disease, and especially around the people affected by it. Gomes was stripped naked and scrupulously examined by the team of doctors just over two months later. They concluded that “the changes seen in the face, trunk, and arms that previously were attacked by tuberculous leprosy are pleasing to the eyes of the physician, since they offer complete hope that if the patient Souza Gomes diligently uses the methods that have been proven, his health may be completely restored” (Interior, 22 fev. 1848, p.3).

In October 1848, Passos the Indian healer came to Belém and was received by the president of the province, the chief of police, a city doctor, and another from the Santa Casa hospital. The president, Jerônimo Francisco Coelho, attempted to persuade him to describe how he produced and used assacu and promised payment in return. Passos assented, but continued “on the assumption that a secret was being taken from him, [a secret] he considered himself to be the owner of and which he applied empirically” (Pará, 1848, p.96). Since colonial times, in Portugal as well as Brazil it was common for the “discoverers” of cures for previously incurable illnesses to keep their formulas to themselves as “secret remedies.” Some holders of these secrets were physicians, surgeons, pharmacists, healers, and also people outside the healing arts (Marques, 1997). Meanwhile, another class of secret holders, the “discoverers and propagators of astonishing remedies” (Santos Filho, 1977, p.356), were generally considered quacks by the representatives of official medicine. Gabriela dos Reis Sampaio (2001) states that charlatanism was a broad category used by medical intellectuals to qualify any and all medicine different from their own, from healers and spirit workers to pharmacists, as well as homeopaths and foreign doctors whose diplomas were not accepted by the country’s medical schools. This author describes doctors as having derived their strength from using the weapons they had available “to not be shipwrecked in this sea of medicines – and to establish their practice as hegemonic” (p.53). Sampaio illustrates how the medical class was marked by internal conflicts and contradictions.^9^

The president of the province subsequently authorized the establishment of a ward exclusively to test assacu on the lepers in the Tucunduba asylum (Pará, 1849, p.60). Since its establishment in 1815, this leper hospital was administered by Santa Casa de Misericórdia of Pará, and throughout the nineteenth century most of the patients interned there were former slaves who had been freed by their owners after exhibiting the first signs of leprosy. Of the 68 patients at the Tucunduba asylum in 1848, 64 were identified as people of color; of these, 51 were identified as slaves and nine as freed (Henrique, 2012).

The first challenge was to find a place suitable for establishing an “experimental leper hospital” where six patients with the disease chosen from Tucunduba would be subjected to these experiments. José Pio de Araújo Nobre of Santa Casa said that “we have been unable to [find a location], because no one wishes to rent for such a purpose” (Ofícios..., 20 dez. 1848), further reinforcement of the stigma that people with leprosy faced during this period. Santa Casa ultimately used a government building in Belém on what was once known as Rua do Atalaia (currently Travessa Joaquim Távora). After adaptations were made, Nobre told the president of the province that the space was ready to receive “the lepers who will undergo the Assacu cure according to the method of the Indian Passos” (Ofícios..., 13 abr. 1849).

The president then determined that Doctor Camillo Guimarães would go to the Tucunduba leper asylum and select “4 patients with all the characteristics of leprosy, in order to join the tests and cure experiments” (Ofícios..., 13 abr. 1849). The concern with choosing patients “with all the characteristics of leprosy” reflected the uncertainties involved in diagnosing leprosy at that time.^10^ The process of naming and describing leprosy during the first half of the nineteenth century was complex and subtle, subject to errors and misinterpretation, issues that “served to obscure and confuse diagnoses of leprosy, and, at the same time, they served to compound the already charged symbolic resonance of the disease and the corresponding force and power of its myriad representations” (Robertson, 2003, p.15). Hasty diagnoses caused the internment of people who were not infected with leprosy in leper colonies and hospitals. Commenting on mortality rates in the Tucunduba asylum during 1844-1849, provincial president Jerônimo Francisco Coelho stated that “the lepers worsened on the side of mortality, which is currently up to 39 per 100, from 33 during the previous year. The 5 counted as cured were not actually lepers” (Pará, 1849, p.54-55). The asylum at Tucunduba was also the destination for people with filariasis, mental illness, smallpox, yellow fever, and epilepsy (Henrique, 2012, p.167). For the year 1848, the president reported that there were 68 patients in the leper asylum and that “those classified as cured are patients with other diseases, whose appearances led to incorrect classification” (Pará, 1848, p.96). These factor explain the president’s concern with correct diagnosis in the lepers who would be subjected to the assacu experiment.

The *Correio Mercantil* newspaper in Bahia published an article listing the names of four people with leprosy who had been part of the experiments involving assacu at Santa Casa de Misericórdia in Pará. They were: “Antonio Hilário Martins, white, single, born in Monte Alegre,” who “has a swollen, opaque, wrinkled face that is repugnant because of the deformity of its features;” Raymundo Gonçalves da Cunha, “white, single, born in this city, son of José da Cunha de Assunção;” “Domingos Manoel, Black, Creole and slave of João Henrique da Silva Lavareda, son of Catarina Maria do Espírito Santo and unknown father” and “Maria do Rosário, Black woman born in Acará parish, slave of Manoel Henrique Dias, daughter of Michelle Francisca and unknown father” (História..., 17 mar. 1848, p.1). Besides their names, the newspapers often published descriptions of diseases these patients had previously, in yet another form of stigmatization that affected the lives of the poor, whether freeborn, freed, or slaves.

Doctor José da Gama Malcher described the effects caused by assacu in the four patients with leprosy as follows: “Soon after taking it they felt general malaise, along with mild tremor, cold extremities, and heat extending from the chest to the face” (História..., 17 mar. 1848, p.1), “some anxiety and desire to vomit” (p.2). Each patient vomited between 10 and 15 times. Some experienced nosebleeds, defecated black blood, and felt as if they were being stung by ants.^11^ The doctor expressed optimism in response to their appearance at the start of treatment, as well as interest in claiming credit for the supposed cure:

As I have established, I do not think I am being rash in hoping that if the improvements continue, the four unfortunate [patients] may be able to once again belong to society, from which they were excluded. And if I can do this, what glory for my province! How many benefits for humankind! And what a triumph for medicine! (História..., 17 mar. 1848, p.2).

Other physicians sought fame by presenting specific formulas for utilizing assacu. An article in the *Anais de Medicina Brasiliense* reported that

the master surgeon Mr. Francisco de Paula Cavalcanti de Albuquerque (of Pará) has just communicated to our editors that through a process of his he has prepared an extract of the milk of assacu, which has proven very valuable when applied to six lepers who are receiving treatment in a private leper hospital (Extrato de assacu, jul. 1847, p.203).

Expressions like “if I can do this” and “through a process of his,” in reference to the doctors’ experiments with assacu, erase the role of the Indian Antonio Vieira dos Passos.

In this way, the experiments to cure leprosy with assacu gained national recognition and “private leper hospitals” appeared in various regions of Brazil. An article in the *O Brasil* newspaper of Rio de Janeiro stated: “No boats arrive from Pará that do not bear news of assacu” (A morfeia!, 15 abr. 1848, p.3). The imperial government requested that the Imperial Academy of Medicine conduct experiments to confirm whether assacu effectively cured leprosy (Brasil, 1848, p.21). The Medico-Homeopathic Academy of Brazil, also in Rio de Janeiro, likewise investigated assacu as a cure for the disease (Publicações..., 10 dez. 1847, p.2).

But others sought to make money off this supposed cure. Another article in the *O Americano* newspaper of Rio de Janeiro stated that “genuine and pure medications made of assacu to cure leprosy can be found exclusively at the home of Joaquim Bernardino Martins Caruncho, the only agent in the Brazilian Empire. The advertiser will present documentation of the efficacy of these medications upon request” (Cura da morfeia, 29 abr. 1848, p.4).

The experiments with assacu called attention to some people in Pará, especially because many physicians in that state corresponded with people and institutions in other areas of the country to report the supposed leprosy cure. The physicians Camillo do Valle, Gama Malcher, and Joaquim Frutuoso Pereira Guimarães became corresponding members of the Imperial Academy of Medicine (Parecer..., out. 1847, p.279). Francisco da Silva Castro, along with Guimarães, joined the contributors to the *Anais de Medicina Brasiliense* (Correspondência particular, jul. 1847, p.204).

As the experiments spread, they generated increasing anxiety about their findings: “assacu is like a spiderweb hanging in the gap of therapeutic experiments in Rio de Janeiro. Miraculous effects against leprosy are expected, as they were expected from guano against this disease, although guano failed” (Remédios novos, out. 1847, p.174). Another article stated: “We all impatiently await the outcome of the experiments conducted at the hospitals in Rio de Janeiro, Bahia, and Pernambuco” (Plantas medicinais, out. 1847, p.218).

The repercussions of Passos’s experiment were so broad that additional tests of the assacu cure for leprosy involving 13 patients were conducted at the São Lázaro Hospital in Lisbon; the doctors concluded that this substance could help improve their condition but did not have the power to cure the disease (Assacu, nov. 1848, p.123; Branco Jr., 1850).

The developments after the Indian Antonio Vieira Passos’s experiment were noted by the renowned Polish-born doctor Pedro Luiz Napoleão Chernoviz, who obtained his medical degree at the school of medicine in Montpellier, France and authored various manuals of popular medicine that circulated widely in nineteenth-century Brazil (Guimarães, 2016). In 1841, he wrote Brazil’s first and most important manual on medical therapies during this period, the *Formulário e guia médico* [*Prescription Vademecum and Medical Guide*] (Chernoviz, 1996). After the 1842 publication of his *Dicionário de medicina popular e das ciências acessórias* [*Popular Medicine and Complementary Sciences Dictionary for Family Use*], he became Brazil’s best-known doctor. In the fifth edition of his dictionary, Chernoviz (1878, p.255) referred to assacu as a “tree from Pará,” the sap and cooked bark of which were “recommended to cure leprosy,” and described in detail the “method to take this remedy (which was used in Pará).” He also noted experiments in Europe to determine whether assacu could cure leprosy: “For some time humankind has believed in the effects of assacu against leprosy, but experiments conducted in Pará and other Brazilian provinces as well as Europe proved that these hopes were exaggerated, and assacu lost the reputation it had as a cure for leprosy” (p.255).

In fact, the tests conducted at “experimental leper hospitals” in Brazil as well as Europe showed that during the first months of treatment assacu led to significant improvements in leprosy symptoms, but then lost this effect. For example, José Joaquim de Souza Gomes himself died roughly two years after he appeared to be “cured” in Santarém (Pará, 1849).

Of the 11 patients with leprosy brought to the experimental leper hospital in Belém, five died, two escaped, three returned to the Tucunduba hospital, and another was interned in the Charity Hospital (Pará, 1848). Assacu’s effects on the body, as described above, help us understand why some patients tried to flee from this treatment. On the other hand, the recurring appearance of supposed cures for leprosy caused people with this disease to occasionally be subjected to similar experiments, all of which had proven unsuccessful up to that point. Recall that Souza Gomes fled to Santarém, in the interior of Pará province, refusing to be interned in the Tucunduba leper hospital. This also occurred in Rio de Janeiro, as we can read in the response of one of the physicians who treated patients with assacu in the leper hospital in that city: “Since I received assacu I began to apply it to various patients in this hospital, but so far only one, who has been the most diligent and docile in following the prescribed treatment, exhibits noteworthy improvements” (Hospital dos Lázaros, 29 abr. 1848, p.3). Not all patients were so willing to be “docile bodies” (Foucault, 2009) in the experiments physicians wished to subject them to.

## Final considerations

After assacu was pronounced unsuccessful for curing leprosy, there was no more mention of the Indian Antonio Vieira dos Passos who reproduced the experiences of his Indigenous ancestors, and without having set foot in a medical school stoked the hopes of people in Brazil and abroad that this plant could contain an effective active ingredient to cure this disease. Did he return to Santarém? A document in the 2nd Hall of Records for Vital Statistics in western Pará offers an important clue. A record of death there noted the following:

On the twentieth of may in 1883 Francisco Vieira dos Passos, resident in the Muratuba *igarapé* and laborer, born in Vila Franca, came to my hall of records and declared that at three o’clock in the morning of that same day in the house where he lived in Muratuba his mother, Francisca (illegible) da Conceição, seventy years of age and married to Antonio Vieira dos Passos, died without a will, leaving five children from this marriage (Óbitos, 1879-1888).

If this was the same Antonio Vieira dos Passos, he married Francisca da Conceição and had five children who lived in Muratuba. Vila Franca, where Francisco Vieira dos Passos was born, is near Santarém, where Passos was imprisoned.

Passos the Indian’s experience shows that the search for a cure for leprosy was not restricted to the field of official medicine. In various parts of the world, healers, shamans, and others classified as quacks conducted their own experiments to cure this disease. I was, by the way, unable to find any document decrying Antonio Vieira dos Passos as a quack or a charlatan, which may be explained by the respect that physicians tended to have for Indigenous medicine and the initial success of the experiment involving assacu. The varied work of healers was associated with “manual labor, low social status, reserved for groups on the margins of society such as slaves, freed men and women, the poor, and women” (Guimarães, 2016, p.25). The people that Antonio dos Passos claimed to have cured of leprosy came from these social strata.

Lepers were left with stigma, the pain of being excluded from social life (especially if they were poor), and all types of trials in “experimental leper hospitals.” But that wasn’t all; as we have seen, some were able to escape or refused to take part in these tests. And those who lived in the leper hospitals and centers did not agree with the vision of these places as “cemeteries for the living” (Henrique, 2012).

Considering the blurred line between official medicine and the healing practices that were popular during the nineteenth century (as we have shown throughout this article), the authorities of Pará province set out to learn the “method of the Indian Passos,” a representative of people who (quite unlike the figures idealized in Brazilian “Indianism”) tended to be defined in the reports penned by the presidents of the province as lazy and the embodiment of the last stages of human degeneration (Henrique, 2003). While Passos’s experiment failed to cure leprosy, many others by Indigenous people were successful and were incorporated into western knowledge in the cures for various diseases. Besides ipecac (*Psychotria ipecacuanha* (Brot.) [Stokes]), used in cough remedies and in a syrup to induce vomiting, and the tonic, digestive, and anti-diarrheic ayapana (*Ayapana triplinervis* [Vahl] R.M. King & H. Rob.), there is also quinine, a substance extracted from the bark of the quina or cinchona tree, discovered by the native people of Peru and used to cure fevers. Quina is a member of the genus *Cinchona*, family Rubiaceae, and was integrated into western medicine to treat malaria; according to Nieto Olarte (2006, p, p.19), it is “the most important and controversial plant from the Americas in the history of medicine.” Another example is andiroba (*Carapa guianensis* [Aublet]), which is widely used in Amazonia as an analgesic, antibacterial, anti-inflammatory, antifungal, and antiallergic and has also been proven effective against wounds, hematomas, rheumatism, and ear infections (Sousa et al., 2019). Meanwhile, physicians attempted in their own way to refine these experiments, translating them into so-called scientific language in order to make them more legitimate and, if they were lucky, immortalize their own names as having discovered a cure for leprosy.

The experiments begun by the Indian Passos in Santarém around 1847 spread around the world, in a circle which in a way closed with Chernoviz’s declaration that assacu’s reputation as a “remedy for leprosy” had reached its end. In this case, we can note that “a world examined through the lens of the history of leprosy shows complex convergences of national histories, international, governmental and medical politics” (Robertson, 2003, p.2). Still, while the experiments with assacu did not start with the Indian Antonio Vieira dos Passos, they also did not end with him. In 1921, a Colombian healer named Mamerto Cortés again promised a cure for leprosy using assacu, and was condemned by the physicians of Belém as a charlatan (Gomes, 2019).

Studies on the history of medicine in nineteenth-century Brazil are essential to recover and reveal the active roles played by Indigenous people in the process of creating and institutionalizing medicine in the country. Besides the labor they provided, Indians in Brazil played a notable role in solidifying western knowledge in fields including botany, agronomy, and medicine. Throughout the nineteenth century, Indigenous knowledge about medicinal plants was widely recognized and utilized by physicians who wanted to incorporate them into their therapeutic repertoire. Assacu was abandoned as a treatment for leprosy, but continued to be used for other purposes. For example, in 1932 Paul Le Cointe (1947, p.55-56) said that “there is no basis to claims that this sap can cure leprosy” but “the infusion of the male flowers (ears) or fresh bracts are applied to boils; the effects are very rapid and [this treatment] should be interrupted as soon as the boil begins to soften. Leaves ground with water are applied for rheumatism.”

While assacu did not produce the desired results in curing leprosy, medicinal uses of many other plants among Indians became part of official medicine, as in the cases of ipecac, ayapana, copaíba (*Copaifera langsdorffii* Desf.), and many others. Recovering these experiments is also a way of recording Indigenous participation in the history of Brazil as well as the history of medicine.

## NOTES

1 According to Erving Goffman (1975, p.7), stigma describes the “situation of the individual who is disqualified from full social acceptance” and consequently subject to an “attribute that is deeply discrediting” (p.13), often used to confirm the “normality” of others. The stigma around leprosy characterizes what this author called “abominations of the body,” in which an individual is rejected in social interactions because of one of his or her attributes, in this case, “physical deformities” (p.14). For studies on leprosy that utilize the notion of stigma in different contexts, see Romero-Salazar et al. (1995); Monteiro (1998); Martins, Caponi (2010).

2 Here I have opted to maintain the terms “leprosy” and “lepers” (and their Portuguese equivalents in the original article), since this was the terminology used in the nineteenth century, thus avoiding an “unacceptable anachronism” (Benchimol, Sá, 2003, p.82). However, it is important to note that Brazilian federal legislation (Brasil, 1995) officially changed these terms (to *hanseníase* and *hansenianos*, corresponding to Hansen’s disease) in order to combat the stigma associated with this disease.

3 Here and for other citations of texts in Portuguese, a free translation has been provided.

4 The article about the supposed cure for leprosy was published on June 9, 1847. Unfortunately, I was unable to find this issue of the newspaper in question. Many of the materials used in this research were published in the *Treze de Maio* newspaper and reprinted by newspapers in other regions of Brazil, and could be searched for in the Brazilian National Library’s Digital Collection.

5 Jack Goody extended the criticism of this dichotomy to the work of Lévi-Strauss, with its division between “wild thinking” and “domesticated thinking.” According to Goody (2012, p.20), Lévi-Strauss was a victim of the ethnocentric binarism embedded into all our categories, even though this may have been through more positively expressed dichotomies. Thanks to Nelson Sanjad and Patrícia Melo for this observation.

6 According to Jane Felipe Beltrão (2004, p.206), ipecac was part of the repertoire of medications utilized by health professionals who worked with the public in Pará, and also appeared on the list of medications that comprised the “domestic pharmacy” considered ideal by Chernoviz.

7 *Pardo* is a term used to denote mixed-race people.

8 On the use of mururé to treat syphilis in colonial Amazonia, see Vieira et al. (2019).

9 On the conflicts between physicians and healers in Amazonia during the nineteenth and early twentieth century, see Figueiredo (1993, 2008a, 2008b); the latter citation contains a discussion linking native healing and leprosy.

10 The bacterium that causes leprosy was only identified in 1873, by the Norwegian physician Gerhard Hansen (1841-1912). From that time onward, with the advent of microbiology (known as the age of bacteriology), understanding of this disease and its etiology changed. The neo-Hippocratic explication that considered issues like temperament, “unrestrained lifestyle,” and the miasma theory gave way to the host-environment-agent triangle.

11 Years later, in a text originally published in 1932, Paul Le Cointe (1947, p.55-56) referred to assacu as a “very caustic and poisonous sap that produces ulcers when it comes into contact “with the mucous membranes and even the skin,” and that “the seeds cause vomiting, constriction of the throat, diarrhea, tenesmus, and fainting.” According to the Indians of the Upper Juruá in the Amazon, “bees avoid a tree called açacu (*Hura crepitans,* Euphorbiaceae) because it is poisonous, and guarantee that they have even seen dead bees on the ground around it” (Almeida, Cunha, 2002, p.618).
